# Robotic Handle Prototypes for Endoscopic Endonasal Skull Base Surgery: Pre-clinical Randomised Controlled Trial of Performance and Ergonomics

**DOI:** 10.1007/s10439-022-02942-z

**Published:** 2022-03-08

**Authors:** Emmanouil Dimitrakakis, Holly Aylmore, Lukas Lindenroth, George Dwyer, Joshua Carmichael, Danyal Z. Khan, Neil L. Dorward, Hani J. Marcus, Danail Stoyanov

**Affiliations:** 1 Wellcome/EPSRC Centre for Surgical and Interventional Sciences (WEISS), University College London (UCL), Charles Bell House, 43-45 Foley Street, London, W1W 7EJ UK; 2 Queen Square Institute of Neurology, University College London (UCL), London, UK; 3 National Hospital for Neurology and Neurosurgery, London, UK

**Keywords:** Medical robotics, Handheld robotics, Robotic-assisted minimally invasive neurosurgery, Endoscopic endonasal skull base surgery, Surgical ergonomics

## Abstract

Endoscopic endonasal skull base surgery is a promising alternative to transcranial approaches. However, standard instruments lack articulation, and thus, could benefit from robotic technologies. The aim of this study was to develop an ergonomic handle for a handheld robotic instrument intended to enhance this procedure. Two different prototypes were developed based on ergonomic guidelines within the literature. The first is a forearm-mounted handle that maps the surgeon’s wrist degrees-of-freedom to that of the robotic end-effector; the second is a joystick-and-trigger handle with a rotating body that places the joystick to the position most comfortable for the surgeon. These handles were incorporated into a custom-designed surgical virtual simulator and were assessed for their performance and ergonomics when compared with a standard neurosurgical grasper. The virtual task was performed by nine novices with all three devices as part of a randomised crossover user-study. Their performance and ergonomics were evaluated both subjectively by themselves and objectively by a validated observational checklist. Both handles outperformed the standard instrument with the rotating joystick-body handle offering the most substantial improvement in terms of balance between performance and ergonomics. Thus, it is deemed the more suitable device to drive instrumentation for endoscopic endonasal skull base surgery.

**Supplementary Information:**

The online version contains supplementary material available at 10.1007/s10439-022-02942-z.

## Introduction

Minimally invasive neurosurgery is benefiting from robotic technology at a much slower rate than other surgical fields, due to anatomical and technical challenges.^15^ One such example of minimally invasive neurosurgery that could be enhanced by robotic technology is the Expanded Endoscopic Endonasal Approach (EEEA).^30^ The EEEA is performed with the use of an endoscope and standard rigid instruments, and aims at the removal of sellar and parasellar lesions, as well as lesions from the regions from the cribriform plate of the anterior cranial fossa to the foramen magnum in the anteroposterior plane.^10^ Although a promising alternative to transcranial approaches, one of the main limitations of this surgical procedure is that standard neurosurgical instruments lack articulation and limit dexterity, and are, thus, making some areas of interest difficult or even impossible to reach.^28^

The enhancement of the EEEA is a popular research field with clinical translation potential. One of its main research disciplines concerns the development of tele-operated robotic platforms. These platforms often employ concentric tube robots (CTR),^6,38,40^ since their small diameter can help reach inaccessible areas inside the constrained surgical workspace at the base of the brain. However, some of the main issues associated with CTRs in surgery are their distal-end dexterity and force-delivery capabilities.^26^ This is why the robotic systems intended for the endonasal approach often employ articulated miniature end-effectors.^1,9^ These systems can also be potentially incorporated into magnetic resonance imaging (MRI) machines, so that the robotic platform can be used simultaneously with image acquisition.^16^

While it is common that the surgical robots developed with the aim to aid surgical approaches, including the EEEA, are tele-operated, they are not the sole robotic device type used in surgery. Another category of surgical robotic devices are handheld surgical robots that can have different advantages when introduced into the operating theatre. These include their smaller footprint and the fact that they can be associated with smaller purchasing and maintenance costs. Additionally, they can be easily integrated into the surgical workflow because they can be interchangeable during the procedure and they often resemble traditional devices which the surgeons already know how to control, reducing, thus, the surgeon’s training period.^27,33^

Combining the advantages of handheld robotic mechanisms with the increased manipulation of CTRs, the first fully-handheld CTR intended for minimally invasive surgery (MIS) was developed.^18^ This device controls the robotic end-effector with a trackball and buttons and preliminary experiments and user-studies showcase its promise, without, however, evaluating its procedural ergonomics. A handheld surgical device that controls an articulated end-effector with a more traditional joystick and trigger setup was compared against a conventional, non-articulated surgical tool,^25^ outperforming the latter in complex surgical training tasks. Despite the improved performance, the majority of the study participants found its design uncomfortable. Alongside these research systems, commercial handheld surgical robots have been introduced into the surgical workflow, such as the Kymerax Precision-Drive Articulating Surgical System (Terumo Co, Japan), a robotic laparoscopic device that has been used in-human for a total hysterectomy.^23^

Following different design approaches, some handheld surgical devices employ more intuitive control methods. Such an example is a robotised needle-holder with a 7 degrees-of-freedom (DoF) handle that allows for wrist control.^17^ This robotic device was evaluated using a force-sensing test platform and did not demonstrate superiority compared to conventional needle-holders after short-term training. A surgical device similar to this design achieves enhanced manipulability by adopting an isomorphic DoF layout and a stable grip force that was produced with a modelling method for grip force pre-compensation.^42^ The feasibility of this device was verified during animal trials with an ergonomic analysis intended for future work.

Alongside robotic surgical devices that achieve enhanced articulation, there are some fully mechanical instruments. Whether they are forearm-mounted,^2^ or finger-operated,^8^ these devices can offer increased manipulation and dexterity. Despite the extensive workspace when compared to non-articulated tools, these purely mechanical devices lack the advantages that robotic instruments offer such as tremor reduction, robotised guidance and incorporation of imaging and sensing.

With the popularity of handheld surgical robotic devices increasing, it is very important that these devices are ergonomically designed since there are significant physical problems related to surgical techniques that could result in discomfort for the surgeon.^37^

To assess the ergonomic design of various robotic instruments, a number of user-studies have been carried out. The robotic needle-holder Jaimy (Endocontrol, France) was compared with a traditional needle holder and showed statistically improved posture under the Rapid Upper Limb Assessment (RULA) in a study involving 14 clinicians.^3^ Two of the most common manipulation methods that handheld robotic instruments employ are joystick- and wrist-control. An assessment of both control methods was carried out with 17 clinicians performing a needle-driving task using both actuation means.^32^ In this particular experiment, the joystick-controlled instrument outperformed the wrist-controlled. Finally, the DEX Robotised Laparoscopic System (Dextérité Surgical, France) was compared with a standard non-articulated needle-holder in a study that involved 6 surgeons.^36^ Even though using the robotised instrument was more time-consuming, it offered better ergonomics of the surgeon’s hand posture. Based on these literature findings, we can assume that it is difficult to achieve both a substantially improved performance, as well as an improved ergonomic posture, when using a handheld surgical robot.

In this study we present two novel ergonomically designed robotic handles for a handheld surgical robotic device with the aim to increase the efficacy of the EEEA. These prototypes have been developed following different design philosophies that literature suggests could lead to an ergonomically designed device. The first design is based on the suggestion that handheld robotic devices for surgery could benefit from intuitive control methods, while the second is an ergonomically designed alternative to traditional handheld robotic devices. The handle prototypes, as well as a standard, non-articulated neurosurgical tool, were paired with a custom surgical training task virtual simulator and a physical setup that resembled the constrained workspace of the EEEA. To evaluate the performance of the handles, as well as to validate their ergonomic design, we designed and ran a randomised crossover user-study. During this experiment, the participants carried out the same simulated surgical training task with all devices, providing insight on the most suitable robotic handle for endoscopic endonasal skull base surgery.

## Materials and Methods

### Development of the Forearm-Mounted Handle (FMH)

**Figure 1:**
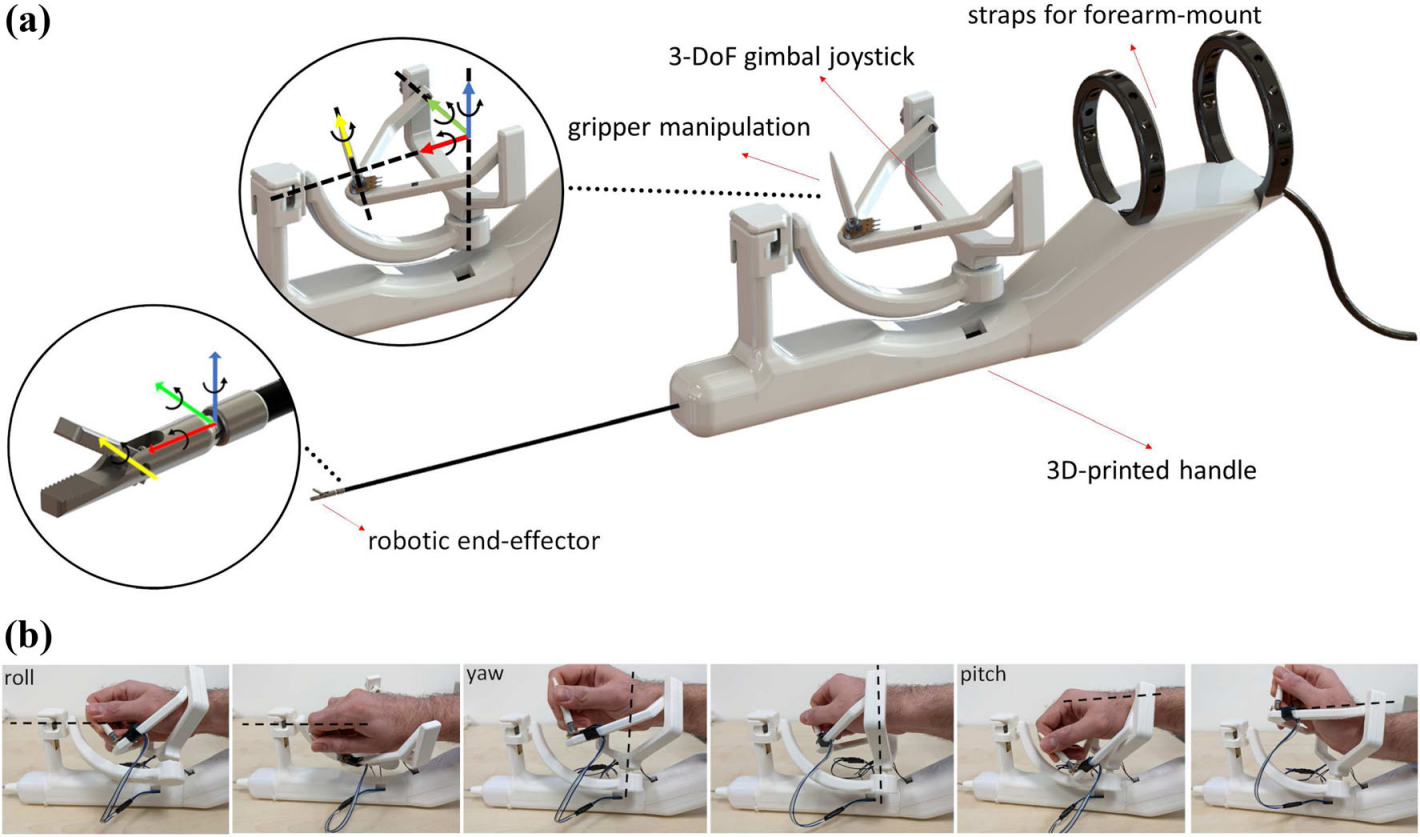
(a) Rendering of the forearm-mounted handle prototype with the coordinate frames of the handle joints and the corresponding coordinate frames of the robot-joints, and (b) The operator’s wrist driving the device in the roll, pitch, and yaw axes.

The robotic end-effector that both the handle presented in this Section, as well as the handle presented in “Development of the Rotating Joystick-Body Handle (RJH)” section, are aimed to manipulate in future work, is a miniature tendon-driven three-DoF robot, with a diameter of 3.6 mm and a length of 1.97 cm. This end-effector has been developed and presented in previous work.^13^ The robotic end-effector alongside its coordinate frame system are shown in Fig. 1a.

This handle prototype follows the ergonomic suggestion that handheld robotic devices could benefit from intuitive manipulation. Its development, as well as the set of literature suggestions that were followed during its design process, have also been presented in previous work.^14^ The handle has maximum dimensions of 42 cm length, 16 cm height, and 431 g weight. It employs a 3-DoF gimbal joystick, with an additional rotating pen-like manipulation DoF for the opening and closing of the gripper. These 4 DoF are directly mapped to the robot-joints pitch, yaw and roll, as well as the gripper DoF to offer easy intuitive control. The handle is forearm-mounted with adjustable straps, in order to alleviate the surgeon from any wrist fatigue and strain.

By mounting the handle on the surgeon’s forearm, the origin of the coordinate axes frame of the joystick coincides with the corresponding origin point on the surgeon’s wrist, creating a stable platform on which the surgeon can easily manipulate the robotic joints. The FMH, alongside the DoF mapping on the robot-joints is depicted in Fig. 1a, whereas the operator’s wrist driving the device so that its manipulability is easier to understand are depicted in Fig. 1b. While the device positioning in the latter figure is not realistic, namely with the device placed on a flat surface, the manipulation principle of the 4-DoF joystick is apparent.

The FMH design was preliminarily evaluated and showed substantially improved performance and procedural ergonomics over the standard instrumentation. However, in that initial study^14^ one single surgeon tested the device and thus, further investigation was needed.

### Development of the Rotating Joystick-Body Handle (RJH)

To cater to a larger set of literature suggestions when it comes to ergonomically designed surgical robotic tools than the ones that were previously satisfied,^14^ the second ergonomic handle design follows a different design philosophy. It employs a rotating joystick-body with the joystick controlling the end-effector joint movements and a standard trigger that actuates the gripper.

When trying to design an ergonomic surgical tool, there is not a universally-accepted consensus on specific components or instructions that make a handle design comfortable to use.^12^ However, relevant literature suggests some instructions that could lead to an ergonomic design. One such suggestion is that since each surgeon considers a different handle size optimal, mostly depending on their hand-size, the device should be indifferent to hand-size.^20^ The preferable handle manipulation type is finger-operated, specifically with the thumb and index finger,^43^ and it is important that the thumb is employed for controlling the robotic joints for manipulation precision.^37^ This can be done by a joystick, rotary switch or other device, while the index finger should actuate a round trigger for the opening and closing of the robotic gripper.^39^

Regarding the geometry of the handle, an improved ergonomic handle shape could include a large palmar grip surface and the combination of precision and turning ability.^21^ In the same study it is also stated that the handle with the shaft should maintain a $45^\circ$ angle. Finally, in another article,^34^ it is suggested that in order for a handle to be comfortable, the instrument at rest should be maintained by a partially open hand, just like the hand is kept at rest.

**Figure 2:**
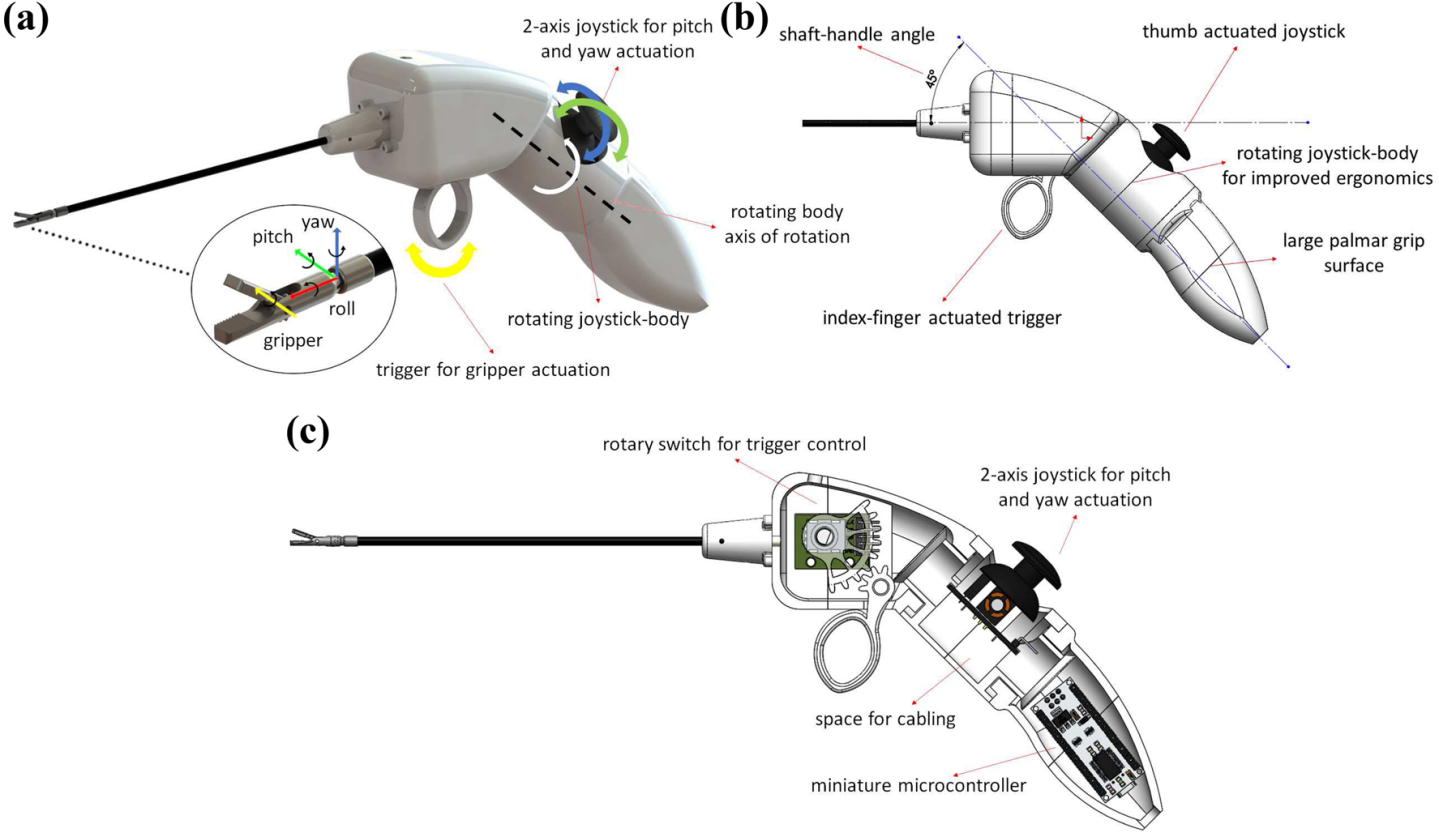
(a) Rendering of the rotating joystick-body handle prototype with the coordinate frames of the handle joints and the axis of rotation of the rotating body (right), and the corresponding coordinate frames of the robot-joints (left). The roll robot DoF is carried out by the surgeon’s hand. (b) The ergonomic specifications analysed in “Development of the Rotating Joystick-Body Handle (RJH)” section reflected on the handle design, and (c) A cross-section sketch of the device that reveals its inner structure.

Following these design specifications, we developed the handle presented in Fig. 2a, with its ergonomic design specifications reflected in Fig. 2b. It is finger-operated, employing a thumb-controlled joystick that actuates the robot joints, namely the yaw and pitch motions since the roll motion is carried out by the surgeon holding the tool, and an index finger-controlled standard trigger that actuates the robot gripper. It contains a large handle surface that provides the surgeon with palmar grip and the handle-shaft angle is $45^\circ$. The robot-joints are controlled by a 2-axis joystick module, while the trigger is controlled by a rotary switch. In this preliminary evaluation prototype, where motors and electronics are absent, the inner structure of the handle consists of the two aforementioned sensory modules, as well as an miniature microcontroller and cabling. A cross-section sketch of the device showcasing these components is presented in Fig. 2c.

**Figure 3:**
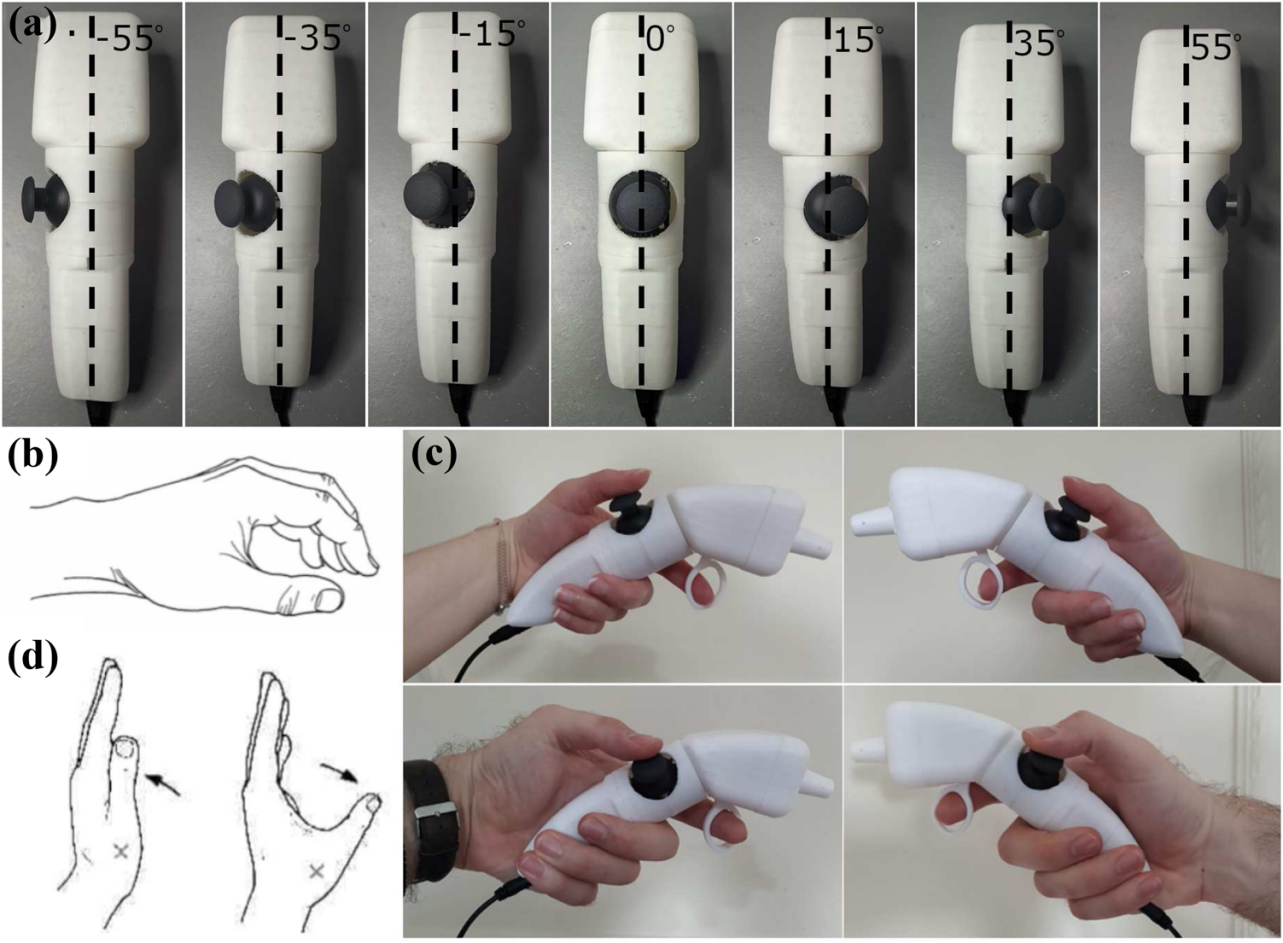
(a) The seven discrete joystick positions on the same 3D-printed rotating joystick-body handle prototype. The axis of rotation of the joystick-body and the angle of each position highlight the rotating function. (b) The hand at its resting position, (c) (left to right and top to bottom) The handle held by a small left hand, a small right hand, a large left hand, and a large right hand, and (d) Thumb adduction (left), thumb abduction (right).

All these suggested ergonomic parameters were accounted for with design modifications. The indifference to the surgeon’s hand-size, and designing the handle in a way that the instrument at rest is maintained by a hand that is also kept at rest, were bigger challenges. To solve both problems, we introduced a rotating joystick-body that is modifiable and can be rotated and tight-fitted into seven discrete positions, in order to be placed at the position that each surgeon feels more comfortable with. Fig. 3a. shows the handle with its rotating body in its different positions. The angles for these positions were $\pm 15^\circ$, $\pm 35^\circ$, and $\pm 55^\circ$, in order to cater to small, medium, and large hands respectively as literature defines them.^22^

The level/resting position of the hand is shown in Figs. 3b and 3d, the thumb is shown in ’adduction’ (left) and ’abduction’ (right). It is evident from this figure that the resting position of the hand requires the thumb to be in an ’abduction’ position.

If we were to place the joystick at the exact centre of the handle, the thumb would be at an ’adduction’ position and the chance that the surgeon would feel uncomfortable and easily tired could be higher. By placing the joystick on a rotating body, the surgeon can rotate the joystick to the left if they are to use it with their right hand and to the right, if they are to use it with their left hand. The angle of rotation, namely the angle by which the surgeon needs to rotate the joystick body to feel comfortable, depends on the surgeon’s hand-size. In Fig. 3c, it is shown how this handle can cater to different hand-sizes and can be used independently of right- or left-handedness. To cater to the smaller hand, the rotating body has been rotated by $\pm 15^\circ$, whereas for the larger hand, the angle was $\pm 55^\circ$.

### Randomised Crossover User-Study Design

The purpose of this study is to expand on the preliminary findings we obtained for the FMH,^14^ to evaluate the newly developed RJH, and to investigate if one of the two is superior. To do that, we organised and ran a randomised crossover user-study where a total of nine medical students used the novel handles and a standard neurosurgical instrument carrying out a surgical training task inside a custom virtual simulator. To conduct the study, we sought and obtained ethics approval by the University College London Research Ethics Committee (UCL REC - reference 18035/001).

The custom virtual simulator was initially presented in previous work^14^ and extended for the needs of this comparative experiment. The simulated surgical training task is a ’peg-transfer’ task, taken from the McGill Inanimate System for Training and Evaluation of Laparoscopic Skills (MISTELS)^11^ because it is indicative of surgical skill when carried out in small and constrained spaces, and it is a task that is highly affected by the lack of articulation.^29^

When designed to adhere to the constrained operative space of the endonasal approach, by manipulating the dimension of the pegs and peg-board, this task can represent the effect that the added articulation can have in keyhole neurosurgery. The peg-board bounding-box dimensions, including the pegs, are $15 \times 30 \times 40$ mm. This box is within the limits of the $30\times 30\times 90$ mm volume that defines a small working space during surgical training that is representative of transcranial approaches.^29^ While we are focused on the endonasal approach, the operative working space and the region within which instruments must operate during transcranial approaches are similar, despite the different access pathways. The pegs were placed all around this volume with some of them purposefully positioned in coordinates where it would be difficult for the standard instrument to reach them, to highlight the importance of articulation.

To prototype both handles we deployed additive manufacturing techniques, namely 3D printing. All parts of the handles were 3D-printed (Ultimaker S5, Ultimaker BV, Utrecht, Netherlands), using polylactic acid (PLA). For the end-effector shaft, a 3 mm diameter stainless steel rod was used, whereas the end-effector was simulated within the virtual environment. The data from the joystick and rotary potentiometers used in both prototypes were processed using a miniature microcontroller (Arduino Nano, Arduino AG, Italy).

Other than the two handle prototypes, additional tools were used for the comparative experiment. These were a 28164TA surgical forceps (Karl Storz SE & Co. KG), and a 3D-printed endoscopic device aimed for camera manipulation by the person carrying out the experiment. During the endonasal approach, a single surgeon can hold the camera in their one hand and the operating instrument in their other hand, a passive endoscope-holder can be used, or an assistant surgeon can hold the endoscope while the operating surgeon is using an instrument in each nostril.^10^ In this study, we decided to replicate the first scenario, with each user-subject of the study manipulating both the prototypes and endoscope, so that they have complete control of the task.

All tools used were optically tracked using a motion capture system (Optitrack V120:trio, NaturalPoint Inc., Canada) and custom marker attachments of negligible weight. The optical markers were placed in positions on the handle body where they would not affect the handling of the instrument. Their physical pose on the handle was transformed in the software environment in relation to the simulated robotic end-effector, and thus the operator manipulation could be realistically replicated inside the simulation environment. To constrain the tools in 3D space, we prototyped a physical constraint which was a model of a cranial CT, modified so that it only includes the areas of interest of the endonasal approach.

**Figure 4:**
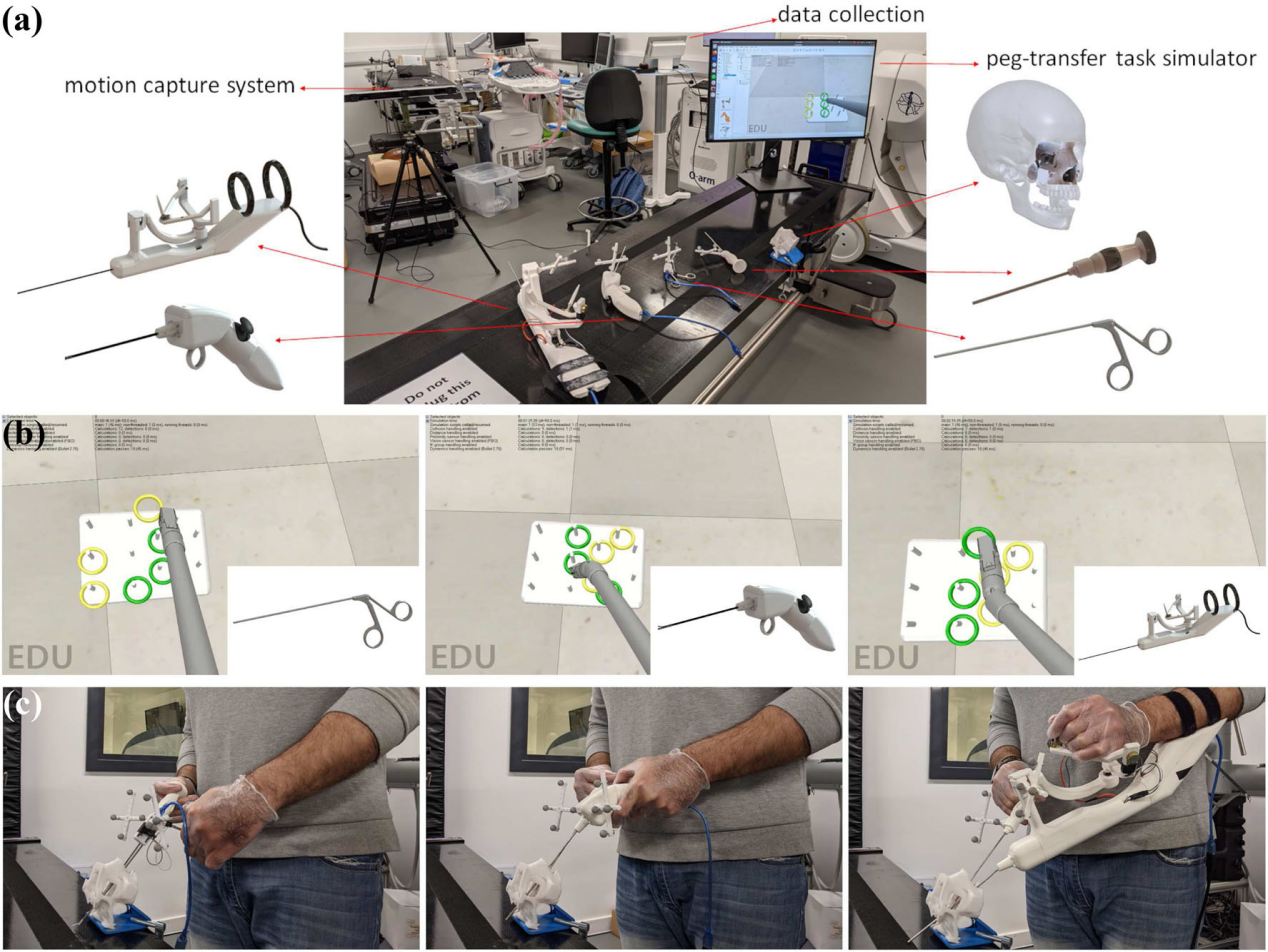
(a) Experimental setup with renderings matched to their respective prototypes. The FMH and RJH are shown on the left side of the image, and the conventional tool that was used as a comparison on the right. Also on the right, the endoscope prototype that was used to manipulate the camera is showcased, alongside the 3D-printed skull constraint. (b) The simulated environment when a researcher is carrying out the peg-transfer task, and (c) The researcher holding the prototypes when carrying out the peg-transfer task.

Finally, the custom simulator was developed in the simulation environment CoppeliaSim (formerly V-REP),^35^ with the same 3D model of the end-effector being used for the two novel handles, while the conventional tool had an end-effector 3D model of the same dimensions, but without the added robotic joint articulation. The experimental setup is shown in Fig. 4a, the simulation environment in Fig. 4b, and the task being carried out by a researcher holding all the tools with their optical markers is shown in Fig. 4c.

The performance and ergonomic assessment of the participants using this universal experimental setup could be highly affected by the individual surgeon’s preferred surgical setup. To alleviate this potential bias, we recruited medical students rather than senior trainees or staff neurosurgeons, that did not yet have an operating preference. A total of nine participants were recruited, with two thirds of them covering the six possible combinations between the three devices. The remaining three participants used the devices in random sequence. The device sequence for each participant is depicted in the Online Appendix A.

At the start of the experiment the participants were asked to complete an adapted version of an already published questionnaire^37^ to access their initial impressions of the three handles. In order to complete this questionnaire, the participants inspected the different prototypes, and briefly used them inside the virtual simulator to get a feel of their operation and control. The aim of this was to assess the face validity of each handle and explore whether there is a general consensus of subjective opinion about each handle between participants. While it is evident that experts would provide better and more formed insight on the face validity of our designs,^4^ having this introductory portion in our comparative experiment proved important, because it gave the participants the opportunity to have a brief trial with the prototypes to understand their operation. The questionnaire that the volunteers were asked to fill in is shown in the Online Appendix B.

Then, the following measurements of participants’ hands were taken: length of the hand, length of the palm, width of the hand at the metacarpal, length of the index finger, width of the index finger-proximal, width of the index finger-distal, and width of the thumb. The purpose of these measurements was to assess if hand-size impacts the performance of each handle.^19^

Using each device in random sequence and whilst wearing surgical gloves, the participants carried out the peg-transfer task. They were maintaining a standing pose and looking at a screen at their eye level, where the simulated task was taking place, and their task was to transfer all rings from one set of pegs to the other, with no particular order. With each device, they attempted the task a total of 10 times, with a maximum duration of 2 min for each attempt. No breaks were taken between these individual 2-min attempts to simulate continuous instrument usage, whereas a 5-min break was scheduled between tool changes. For each attempt, the time at which each ring was successfully transferred was manually recorded, something that can give insight on the completion and success rates, as well as the learning curve of the handles.

To investigate the procedural ergonomics, the participants were observed and assessed while carrying out the tasks, completing the Rapid Upper Limb Assessment, a validated measure to assess the ergonomics of instruments.^31^ When using this score-based system, lower RULA scores for a procedure are associated with better ergonomic postures. The RULA ergonomic scores throughout the experimental procedure with each device were taken at the participant’s worst demonstrated posture.

At the end of the task with each device, the participants were asked to complete the Surgery Task Load Index questionnaire.^41^ This questionnaire assesses the mental, physical, and temporal demands of using an instrument, along with situational stress and distractions during the task. Each participant completed this two-part questionnaire for each of the three devices, and based on their answers 6 weighted SURG-TLX dimension scores for each one of them, and for each device are extracted. The aim of this questionnaire is to assess the ergonomics of the handles from the participants perspective, alongside the more objective Rapid Upper Limb Assessment.

## Results

### Evaluation and Preference Questionnaire

For the first question of the ’Handle Evaluation’ questionnaire, the participants were asked to rate the two handles on four different categories as shown in the Online Appendix B. Overall, the participants preferred the RJH, which scored mean ratings of 4.1 for intuitiveness, 4 for comfort, 3.8 for precision and 3.8 for stability, with the respective ratings for the FMH being 3.7, 3, 3.6 and 3.8. This preference was also reflected in the ’Handle Preference’ part of the questionnaire, where the majority of the participants agreed that the RJH felt easier to use, was less tiresome, and it employed easier gripper control. To improve the design of the handles, it was suggested that the RJH incorporates a clicking joystick rather than a standard trigger, while the FMH would feel better with an articulated trigger or button controlling the trigger, rather than the pen-like rotating trigger that controls it in this current iteration.

The hand measurements a.-g. as mentioned in “Randomised Crossover User-Study Design” section taken during this part of the experiment are shown in the Online Appendix C. The participants’ hands were split into categories based on hand length, and following the definition that a hand is considered small when its length is between 16.3 and 17.9 cm, medium when its length is between 17.9 and 19.4 cm, whereas for lengths between 19.4 and 21.2 cm, the hand is considered large.^22^ Thus, the study included five participants with small-sized hands, three with medium-sized hands, and one participant with large-sized hands. Resultantly, the respective angles by which the rotating body on the RJH was rotated based on hand-size category were $15^\circ$, $35^\circ$ and $55^\circ$.

### Performance Evaluation

**Figure 5:**
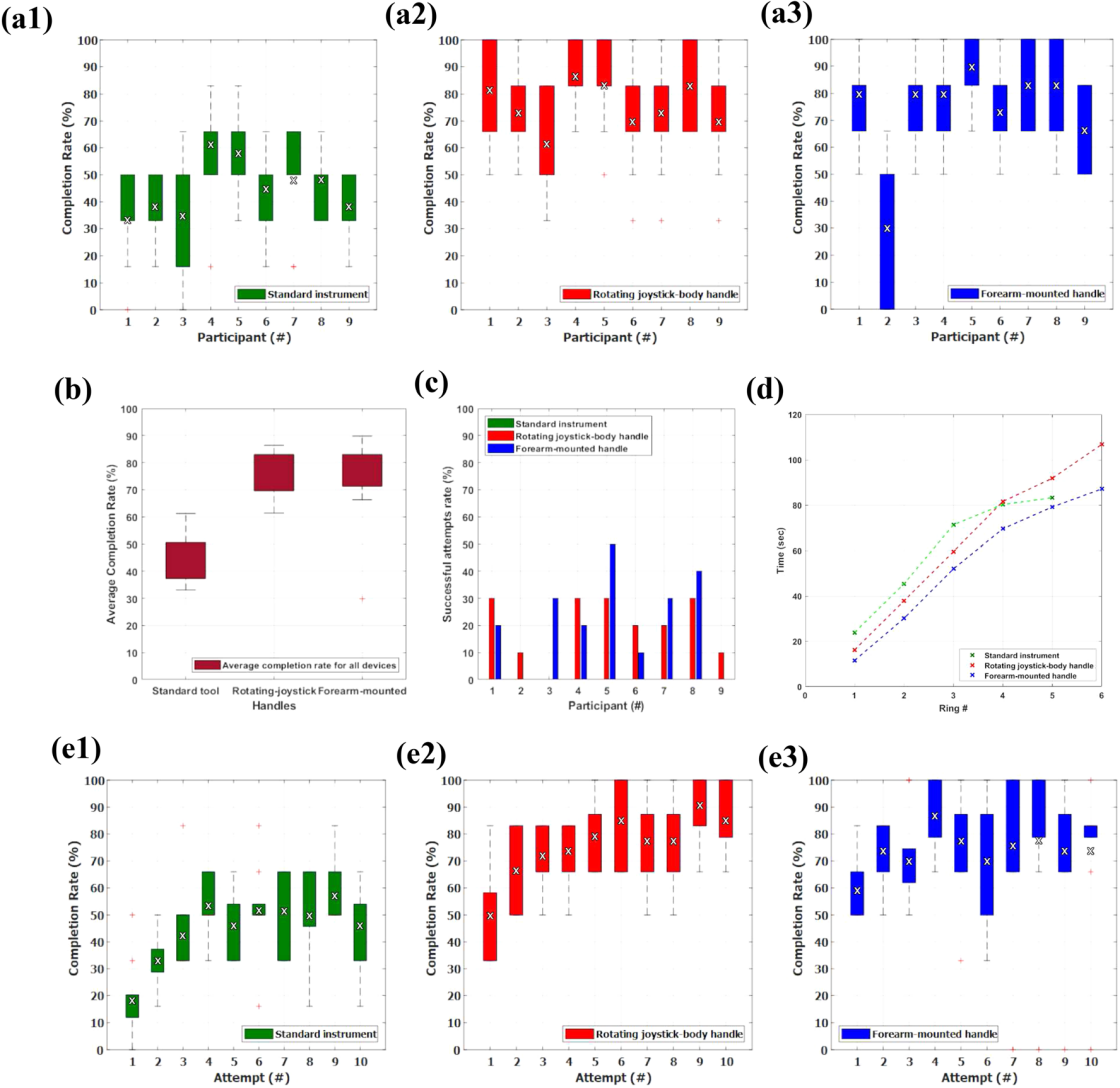
(a) The completion rate during all 10 attempts for each of the 9 participants, for (a$_1$) the conventional tool, (a$_2$) the RJH, and (a$_3$) the FMH, (b) The mean completion rate for all 9 participants for each of the 3 devices, (c) The successful attempt rate for each of the 9 participants, and for each of the 3 devices, (d) The mean time it took all participants to successfully transfer each ring, and (e) The learning curve of (e$_1$) the conventional tool, (e$_2$) the RJH, and (e$_3$) the FMH, presented as the relationship between the completion rate for all 9 participants for each of the 10 attempts. The red crosses in sub-figures (a), (b), and (e). are the outliers of the box-plots, while the black and white x-marks in sub-figures (a), and (e) are the mean average values of the completion rate per participant and the completion rate per attempt respectively.

The completion rate, here defined as the percentage of rings that were successfully transferred from the one set of pegs to the other out of the total of 6 rings, for each of the 9 participants throughout their 10 2-min attempts, and for each of the 3 devices is depicted in Figs. 5a$_1$–5a$_3$. In the same set of figures, the mean average values of the completion rate per participant is shown. The individual completion rate measurement points per attempt, and for each of the 9 participants, can be seen in detail in Online Appendix D for the conventional instrument, in Online Appendix E for the RJH, and finally in Online Appendix F for the FMH. While the use of box-plots makes it easier to understand how the completion rates for each participant are laid out during the experiment, individual measurements can be valuable in order to more thoroughly investigate each participant’s performance.

The median of rings transferred between the 9 participants when they were using the standard instrument was 4, whereas for both the RJH and FMH was 6. With the standard instrument, 2 participants did not manage to transfer any rings at all at least once during their 10 attempts. The least amount of rings that were transferred with the RJH during a single attempt were 2, and with the FMH 3, with the exception of participant #2, who asked to end the experiment early due to shoulder discomfort, as discussed in “Discussion” section.

Overall, the participants showcased significantly improved performances with the robotic handles rather than with the standard instrument, with both the lower and upper quartiles of their box-plots scoring higher for the two novel handles. The mean completion rate, here defined as the arithmetic average percentage of rings that were successfully transferred from the one set of pegs to the other out of the total of 6 rings, between all 9 participants, and for each of the 3 devices is depicted in Fig. 5b. It is evident that the two handles clearly outperformed the standard instrument. Figure 5c shows the successful attempt rate, defined as the percentage of attempts out of the 10 attempts when a participant was able to transfer all 6 rings. Once again, we notice the same trend of the two handles being superior to the standard instrument that had a $0\%$ successful attempt rate throughout.

To have an understanding of the time-efficiency of each handle, we calculated the mean time it took the participants to transfer each one of the 6 rings. The results are shown in Fig. 5d. The participants had the most time-efficient performance using the FMH, and managed to complete the task in a mean time of 87.1 s, almost 20 s faster than when using the RJH that had a mean time of successful completion of 106.7 s.

Finally, in Figs. 5e$_1$–5e$_3$, we quantify the learning curve of the devices as the relationship between the completion rate for all 9 participants during each of their 10 attempts. In the same set of figures, the mean average values of the completion rate per attempt is shown. Once again, for a more thorough investigation, the individual measurement points of completion rate per participant for each of the 10 attempts, are shown in Online Appendix G for the conventional instrument, in Online Appendix H for the RJH, and finally in Online Appendix I for the FMH. When the participants were using the standard instrument, and by the 4th attempt, they were confidently achieving 4 rings, a pattern that stayed mostly the same by the end of the experimental session. On the contrary, when participants were using the RJH and the FMH, they achieved completion rates of over $80\%$ early, and after the 5th attempt they were regularly successfully completing the task, suggesting that the novel handles have small learning curves. Between the two handles, the RJH seems to present a more consistent learning curve with less noise in each attempt compared to the FMH.

### RULA Ergonomic Assessment

**Figure 6:**
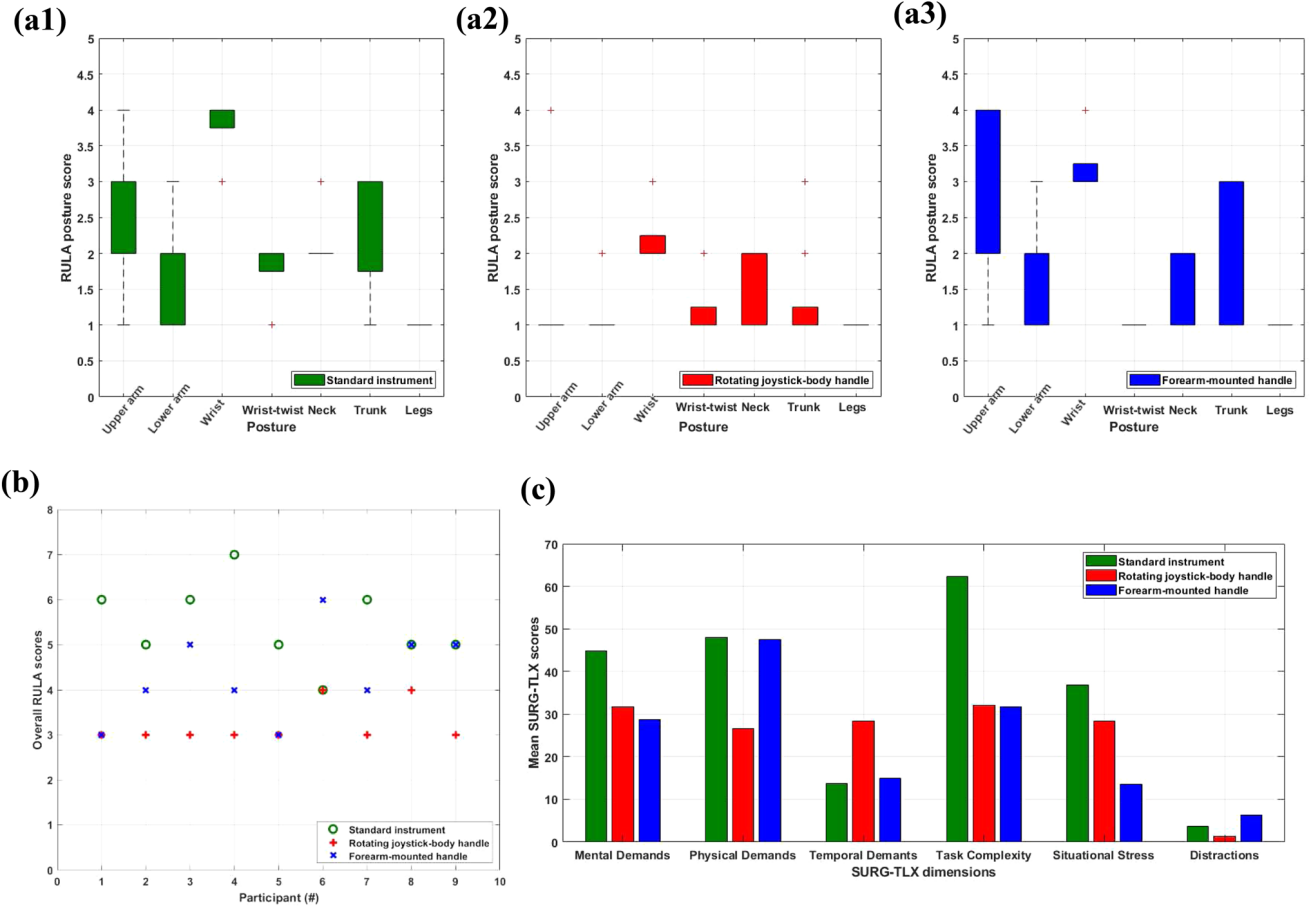
(a) The RULA posture scores for all 9 participants for each posture, and for (a$_1$) the conventional tool, (a$_2$) the RJH, and (a$_3$) the FMH, (b) The overall RULA score for each participant, and for each of the 3 devices, and (c) The mean SURG-TLX scores for each individual SURG-TLX dimension, and for each of the 3 devices.

The RULA survey method posture scores for all 9 participants, and for each individual posture of the upper limbs, neck, trunk, and legs are shown in Figs. 6a$_1$–6a$_3$, whereas in Fig. 6b, these scores are used to calculate the overall RULA ergonomic score for each individual participant when using each device. Compiling these individual posture scores into overall RULA scores for each participant, the RJH had a mean RULA score of 3.2, followed by the FMH with an mean score of 4.3, and the standard instrument that scored 5.4.

The upper arm posture was worst for the FMH, where the participants had to raise their shoulders to translate the simulated robotic end-effector, followed by the standard instrument, and then by the RJH. The lower arm posture was similar for the first two devices with the participants occasionally working across the midline of their bodies, and again best performance occurred with the RJH.

As expected, wrist posture was worse with the standard instrument, with extreme angles occurring and participants having to bend their wrist away from the midline. When using the FMH, participants needed to often employ mid-range and extreme angles, while bending their wrist, and when using the RJH they were mostly within a healthy angle range. The final parameter for the RULA posture group A, which includes the arms and wrists, was the wrist-twist, that was almost always near the end of the twisting range for the standard instrument, mainly mid-range for the RJH, and always at the natural wrist twist position for the FMH.

As far as the RULA group B is concerned, namely the neck, the trunk and the legs, participants seemed to struggle more to find a comfortable neck position when using the standard instrument, and they would often twist their neck. The corresponding behavior with the two novel robotic handles was similar between each other, and slightly better than with the standard instrument. When using the RJH the participants would mostly maintain a well-supported trunk and would rarely flex forward, twist or bend it. The same cannot be said for the other two devices, where the participants showed similar behavior that included all flexion, twisting and bending. Finally, throughout the experiment the participants’ legs and feet were well supported and in an evenly balanced posture, meaning that no participant scored more than a score of 1.

### Surg-TLX Questionnaire

The final measure to assess the participants’ ergonomic behaviour was the SURG-TLX questionnaire, a subjective questionnaire that aims to assess the participants’ mental, physical, and temporal demands while using an instrument, alongside with situational stress and distractions during the task.

The mean of the weighted SURG-TLX scores, for each dimension and for each device are shown in Fig. 6c. Summing up all the individual scores for each participant, we can calculate the total SURG-TLX workload for each participant and for each device, shown in Table 1. The smaller the workload, the more favorably in terms of ergonomics the device has been perceived by the participant. The subjective opinions of all 9 participants about their own perceived ergonomics, agree that the two novel handles impose a smaller workload than the standard instrument, with a single exception. Participant #6 preferred the standard instrument over the FMH. Out of 9 participants, 6 preferred the FMH, and 3 preferred the RJH. Finally, and on average, the former outscored the latter in terms of mean total SURG-TLX workload.

**Table 1:** The total SURG-TLX workload for each participant, and for each of the 3 devices, as well as the mean SURG-TLX workload for each device.

Participant #	Total SURG-TLX workload		
	Standard instrument	Rotating joystick-body handle	Forearm-mounted handle
1	230	186	99
2	205	10	197
3	209	192	157
4	188	124	127
5	236	123	124
6	221	212	223
7	171	160	132
8	193	187	142
9	230	143	81
Average	209.22	148.55	142.44

## Discussion

In this study, we presented a novel ergonomically designed surgical robot handle intended for a handheld robotic instrument for endoscopic endonasal skull base surgery, and compared it with a previously developed handle with different design philosophies. The previously developed handle is forearm-mounted with surgeon-wrist to robot-joints movement mapping, and the novel handle employed a rotating joystick-body that can be maintained at the surgeon’s natural resting hand position. To decide which one of the two handle prototypes would be the most suitable to improve the efficacy and human factors of the endonasal approach, we designed and carried out a randomised crossover user study where these robotic handle prototypes were evaluated with a virtual surgical task simulator.

In terms of performance and efficacy, the two robotic handles clearly outperformed the standard neurosurgical instrument. Although an expected result, since a tool with added articulation is designed to have a larger workspace than a non-articulated instrument, performance was still an important aspect to investigate. The ergonomic design considerations implemented in the two designs, namely the forearm constraint of the one handle, and the moving joystick-body of the other, could potentially lead to declined performance because of dexterity loss or imprecise control. The fact that both handles outperformed the standard tool suggests otherwise.

When comparing the performance of the two novel handles, the two devices had very similar behaviours. The RJH showcased a slight edge in the categories of completion rate per participant and mean successful attempt rate, while the FMH performed slightly better in terms of mean completion rate. These differences, however, are small and thus are not adequate to confidently suggest which one of the two novel devices is the most suitable for the EEEA.

Both handles seem to have similar and small learning-curves, with the RJH being associated with a more consistent learning curve, and the participants were able to perform very well or even complete the task very early in their 10 attempts sequence. The one category where one handle clearly outperforms the other is time-efficiency, with participants being able to successfully complete the task using the FMH approximately $22\%$ faster than with the RJH. A final note on performance is that a correlation between hand-size and handle performance could not be immediately identified since participants of all three hand-sizes had similar outcomes in terms of completion and successful attempt rate.

Much clearer conclusions can be drawn from the ergonomic assessment. The RJH is the safest handle to use for an extended amount of time according to the RULA survey, since it only scored 3 and 4, both scores falling into the ’low risk’ category. On the contrary, the FMH scored both 3 and 4, but also 5 and even 6 once, deeming it low to medium risk. As far as the standard instrument is concerned, and with the exception of one ’low risk’ score and one ’high risk’ score, it was deemed medium risk.

Finally, when participants were able to voice their opinion at the start of the experiment, the majority preferred the RJH. This consensus was inverted after the experimental procedure took place with the majority of the participants favoring the FMH in terms of total mental, physical and temporal workload. This preference contradicts the RULA assessment outcomes, with the most probable explanation being that the participants highly valued the intuitiveness of the handle, but only used it for a limited amount of time. Had they used the handle for an extensive period, the RULA assessment suggests that there is a higher likelihood for discomfort.

Despite the low mental demands and situational stress associated with the FMH, as well as its time-efficiency, the poor ergonomic results and the similar performance with the RJH, combined with the increased time and effort it would take to switch between instruments during the endonasal approach, indicate that the more favorable handle for the EEEA, amongst the two investigated in this study, is the RJH. The increased time and effort would be evident in instances when non-robotic tools would need deploying for the operation. To un-mount the FMH from the surgeon’s forearm, the operating surgeon would need to leave whatever tool they hold with their other hand to untie the straps, or a second clinician would need to be involved. With the RJH, on the other hand, the operating surgeon would just need to leave the tool on the operating tray and pick-up another tool. We believe that the RJH device offers a more complete solution than the FMH with similar increased joint articulation, while simultaneously maintaining a low ergonomic risk.

While these findings showcase potential for improved neurosurgical instrumentation, there are still some aspects of this work that need addressing. During the study design, it was decided that the operator would control both the instruments and endoscopic device. While camera control does not immediately affect the perceived ergonomics of each device, since the mock endoscope and instrument did not collide inside the operative workspace, it could affect the performance of the operator. The more time the operator would spend in manipulating the camera, the better perception of depth inside the simulated environment they would develop. To try and alleviate this risk, we deployed randomized tool sequences between the participants. However, different camera setups could showcase interesting results and their exploration could be scope of future work.

Another limitation of our study design, was the decision to not include expert neurosurgeons in the experiment. The reason why we wanted to avoid mixed cohorts for this specific comparative experiment between all three instruments, was that the preferred surgical setup that expert surgeons have developed over the years, would probably lead to a non-representative performance when using the conventional tool, introducing bias to the comparative experiment. However, understanding how helpful the opinion of an expert neurosurgeon cohort would be towards our device development, as part of future work, a human-factors workshop consisting of expert neurosurgeons will be organised, that will validate the performance and ergonomic behavior of our fully-functional handheld prototype.

One of the main points of concern was that despite the fact that most participants preferred the FMH during their SURG-TLX evaluation, one participant could not manage to finish the part of the experiment with this handle due to shoulder discomfort. This happened even after the surgical table was lowered as to fit the participants height, and suggests that this forearm-constraint can be affected by the surgical setup, and thus will not always be suitable depending on the surgeon’s preference and also the operating theatre arrangement.

The same participant evaluated the RJH an order of magnitude lower than the other participants in the SURG-TLX scale. If we omit this outlier value from the average, the FMH would score significantly lower than the RJH. More specifically, the average total SURG-TLX score for the RJH would be 165.87, while for the FMH would be 135.62. While the difference between the two scores would be much clearer, the outcome of this part of the experiment remains the same, ie the participants preferred the FMH over the RJH. Still, the objective RULA evaluation suggests that the chance of them feeling discomfort would be higher with the FMH than with the RJH were they to use the robotic instrument for an extended amount of time.

Operative times for endonasasl approaches vary depending on the complexity of each case and the surgeon experience, ranging from one to 2 h for routine cases,^24^ up to more than 10 h for more complex ones.^5^ A variety of different tools are used for each stage of the procedure, with frequent tool changes.^7^ While we believe that each 20-min session with each handle would be sufficient to give us representative information on ergonomics about shorter approaches with frequent tool changes, more complex procedures would necessitate more elaborate phantoms, tasks, and also the allocation of time from surgeons.

As far as the rotating joystick-body handle is concerned, the design presented in this paper will have to be altered to incorporate motor electronics as well as the robotic end-effector. It will, thus, have to be re-validated for its ergonomics, because while the weight distribution of the functional device will be similar to the suggested prototype, the weight itself will increase with the addition of motors and cables, and the geometry will have to be amended.

In future work, the superior handle prototype will be paired with the previously developed miniature robotic end-effector in order to form a fully functional surgical robotic instrument. This will require redesign in order to fit electronics and actuation tendons. After the new design will have been validated for its improved ergonomics, it would be beneficial to compare its ergonomic behavior with other handheld surgical robotic systems that are commercially available. We also intend to incorporate sensing and feedback mechanisms such as tactile-feedback or imaging modalities. As a final step in the device development, this robotic prototype will be evaluated through surgical training tasks and phantom trials in order to validate its potential.

## Supplementary Information

Below is the link to the electronic supplementary material.

Supplementary file1 (PDF 1171 kb).
