# Supplementary material for: Robotic Handle Prototypes for Endoscopic Endonasal Skull Base Surgery: Pre-clinical Randomised Controlled Trial of Performance and Ergonomics
Source: Ann Biomed Eng. 2022 Mar 8;50(5):549–63. doi: 10.1007/s10439-022-02942-z (PMC9001398; doi:10.1007/s10439-022-02942-z)
Supplement: Supplementary file 1 — Supplementary file1 (PDF 1171 kb). [file 10439_2022_2942_MOESM1_ESM.pdf]

Appendix

A The device sequence for each participant

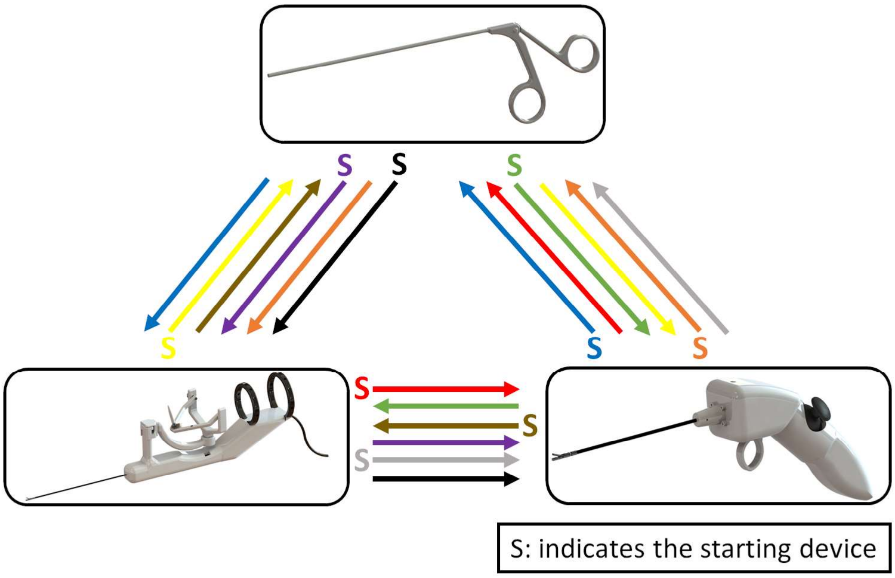

Figure 1: The device sequence for each participant, with each participant being represented by a different color.

B The handle evaluation and preference questionnaire

Handle Evaluation

1. Please look at and touch the handles. Hold each handle, and operate the joysticks and triggers of each one. Then, please indicate a score on a scale of 1 (strongly disagree) to 5 (strongly agree), indicating how much you agree each handle was intuitive, comfortable, precise, and stable.
   a. Rotating joystick-body handle
      i. Intuitiveness:
      ii. Comfort:
      iii. Precision:
      iv. Stability:
   b. Forearm-mounted handle
      i. Intuitiveness:
      ii. Comfort:
      iii. Precision:
      iv. Stability:

2. Would you like to have any additional commands and/or triggers on the handle?
   a. Rotating joystick-body handle: Yes/No

   b. Forearm-mounted handle: Yes/No

Handle Preference

1. Please indicate the instrument handle that you prefer:
   a. Rotating joystick-body handle
   b. Forearm -mounted handle

2. Please could you briefly explain why you prefer this instrument handle:

3. If you have any additional comments about any other handles, please provide them here:

Figure 2: The handle evaluation and preference questionnaire that the participants were asked to fill-in after they inspected the devices and before carrying out the tasks with them.

C The hand measurements of the participants

	Participant #								
**Hand measurement**	**1**	**2**	**3**	**4**	**5**	**6**	**7**	**8**	**9**
**a.**	19	16.5	18	17.5	17	16.5	16.5	20	18.5
**b.**	10.5	10	10.5	10	10	9	10	11	10
**c.**	8.5	7.5	8	8	8	7.5	7	9	8.5
**d.**	7.5	7.4	7	8	7	7	7	9	7.5
**e.**	2	2	2	1.5	1.5	1.5	1.25	1.75	1.5
**f.**	1.5	1.7	1.6	1	1	1	1	1.5	1.25
**g.**	2	1.5	2.3	2	1.75	1.75	1.5	2	2

Figure 3: The participants' hands measurements with a. length of the hand, b. length of the palm, c. width of the hand at the metacarpal, d. length of the index finger, e. width of the index finger-proximal, f. width of the index finger-distal, and g. width of the thumb.

D Individual completion rates per attempt, for each participant

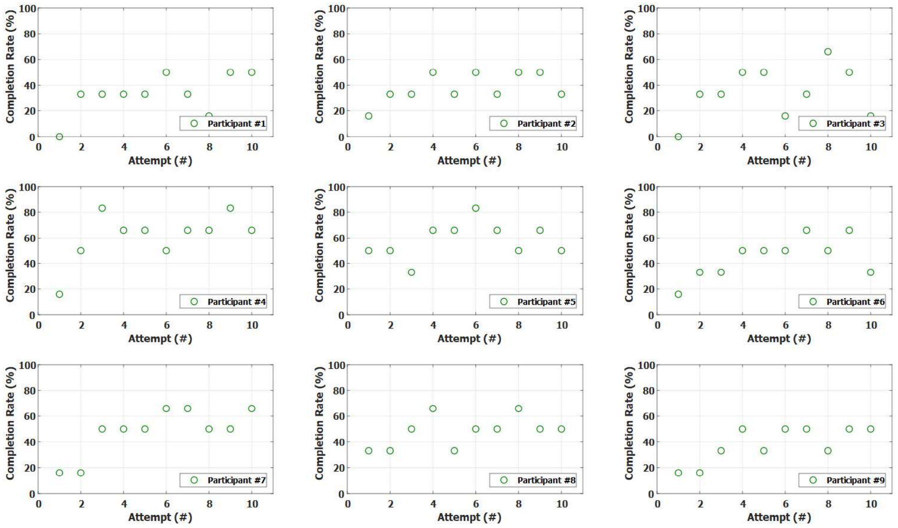

Figure 4: Individual completion rate measurement points per attempt for each of the 9 participants for the conventional instrument.

E Individual completion rates per attempt, for each participant

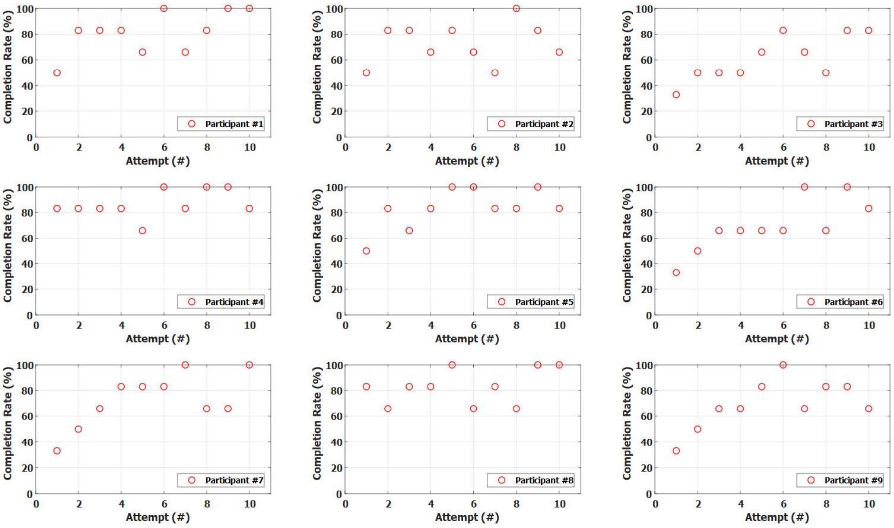

Figure 5: Individual completion rate measurement points per attempt for each of the 9 participants for the RJH.

F Individual completion rates per attempt, for each participant

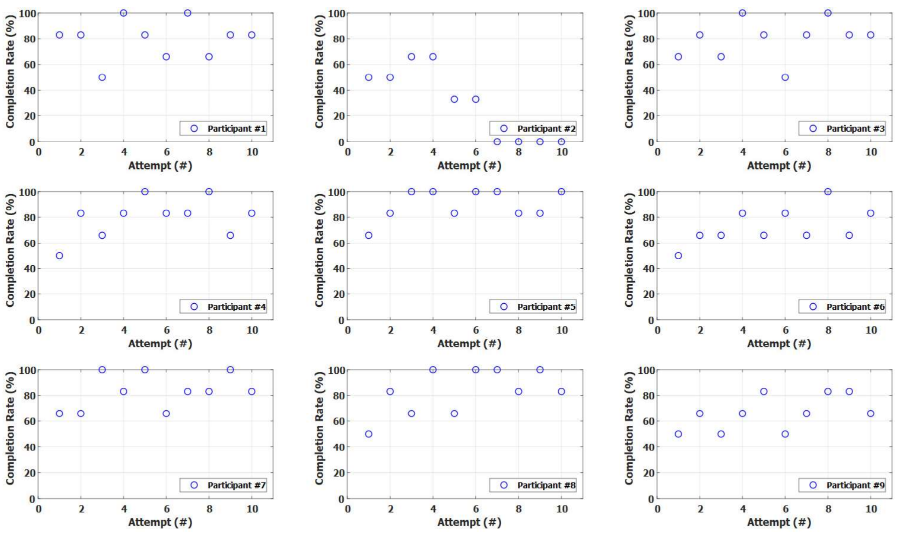

Figure 6: Individual completion rate measurement points per attempt for each of the 9 participants for the FMH.

G Individual completion rates per participant, for each attempt

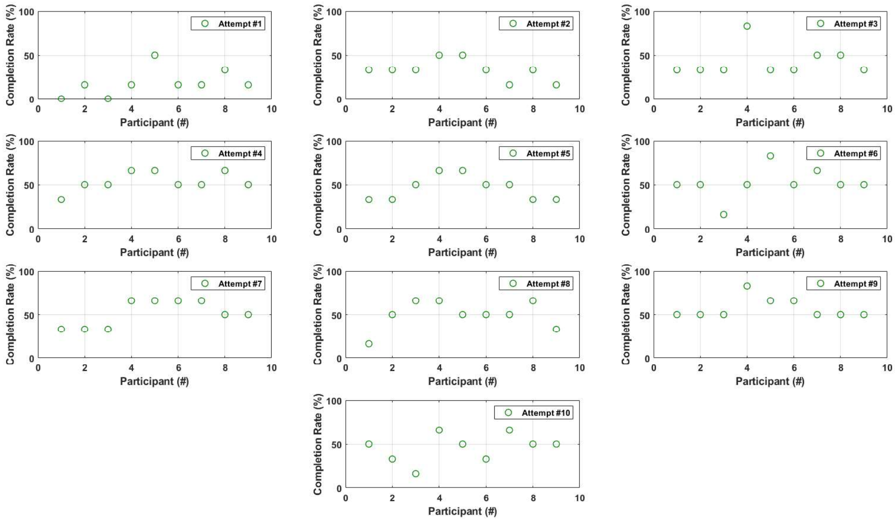

Figure 7: Individual completion rate measurement points per participant for each of the 10 attempts for the conventional instrument.

H Individual completion rates per participant, for each attempt

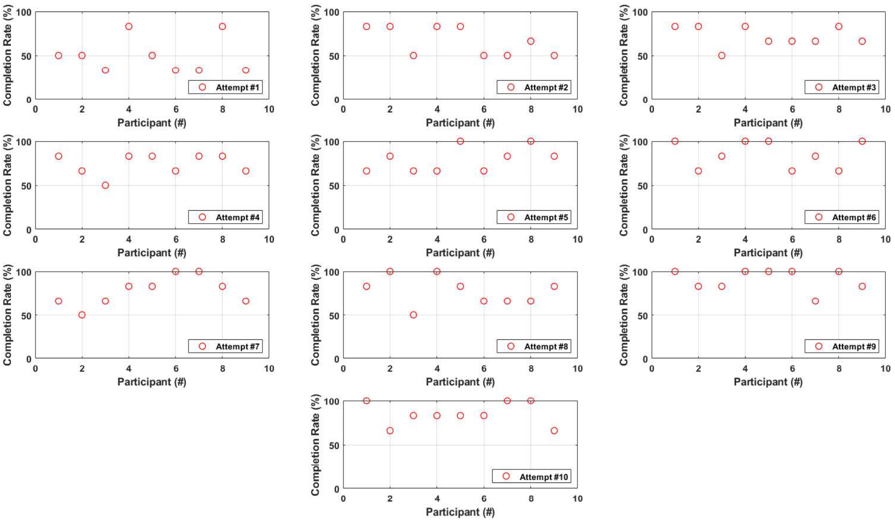

Figure 8: Individual completion rate measurement points per participant for each of the 10 attempt for the RJH.

I Individual completion rates per participant, for each attempt

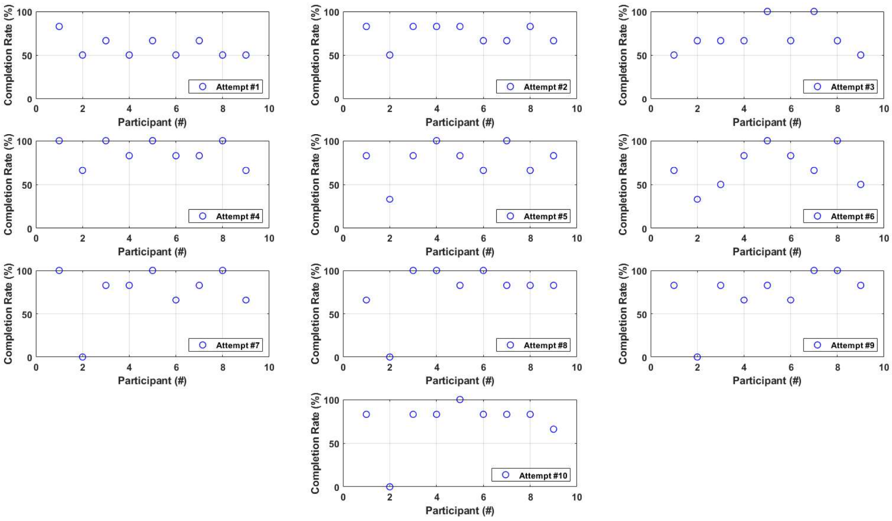

Figure 9: Individual completion rate measurement points per participant for each of the 10 attempt for the FMH.
